# Tractography‐Based Ventral Intermediate Nucleus Targeting: Novel Methodology and Intraoperative Validation

**DOI:** 10.1002/mds.26633

**Published:** 2016-05-23

**Authors:** Francesco Sammartino, Vibhor Krishna, Nicolas Kon Kam King, Andres M. Lozano, Michael L. Schwartz, Yuexi Huang, Mojgan Hodaie

**Affiliations:** ^1^ Division of NeurosurgeryToronto Western Hospital and University Health NetworkTorontoCanada; ^2^ Division of Neurosurgery, Department of SurgeryUniversity of TorontoTorontoCanada; ^3^ Division of NeurosurgerySunnybrook HospitalTorontoCanada; ^4^ Physical Sciences PlatformSunnybrook Research InstituteTorontoCanada

**Keywords:** essential tremor, functional neurosurgery, stereotactic targeting, tractography, ventral intermedius nucleus

## Abstract

**Background:**

The ventral intermediate nucleus of the thalamus is not readily visible on structural magnetic resonance imaging. Therefore, a method for its visualization for stereotactic targeting is desirable.

**Objective:**

The objective of this study was to define a tractography‐based methodology for the stereotactic targeting of the ventral intermediate nucleus.

**Methods:**

The lateral and posterior borders of the ventral intermediate nucleus were defined by tracking the pyramidal tract and medial lemniscus, respectively. A thalamic seed was then created 3 mm medial and anterior to these borders, and its structural connections were analyzed. The application of this method was assessed in an imaging cohort of 14 tremor patients and 15 healthy controls, in which we compared the tractography‐based targeting to conventional targeting. In a separate surgical cohort (3 tremor and 3 tremor‐dominant Parkinson's disease patients), we analyzed the accuracy of this method by correlating it with intraoperative neurophysiology.

**Results:**

Tractography of the thalamic seed revealed the tracts corresponding to cerebellar input and motor cortical output fibers. The tractography‐based target was more lateral (12.5 [1.2] mm vs 11.5 mm for conventional targeting) and anterior (8.5 [1.1] mm vs 6.7 [0.3] mm, anterior to the posterior commissure). In the surgical cohort, the Euclidian distance between the ventral intermediate nucleus identified by tractography and the surgical target was 1.6 [1.1] mm. The locations of the sensory thalamus, lemniscus, and pyramidal tracts were concordant within <1 mm between tractography and neurophysiology.

**Interpretation:**

The tractography‐based methodology for identification of the ventral intermediate nucleus is accurate and useful. This method may be used to improve stereotactic targeting in functional neurosurgery procedures. © 2016 The Authors. Movement Disorders published by Wiley Periodicals, Inc. on behalf of International Parkinson and Movement Disorder Society

Essential tremor (ET) is a common movement disorder with a prevalence of between 0.4% to 4%.^1^ It can significantly compromise a patient's ability to perform activities of daily living.^2^ Effective medications for tremor control are limited.^3^ Surgical treatment for advanced refractory tremor includes lesioning of the ventral intermediate nucleus (VIM) with radiofrequency,^4^ gamma knife,^5^ magnetic resonance–guided focused ultrasound (MRgFUS),^6^, ^7^ and VIM deep brain stimulation (DBS).^3^, ^4^ The precise stereotactic targeting of VIM is associated with improved surgical outcomes.^8^ Image‐based target identification is becoming important for radiosurgery and MRgFUS because little or no intraoperative mapping is possible. However, current magnetic resonance imaging (MRI) sequences (both 1.5 and 3 Tesla) fail to visualize the VIM, and conventional surgical targeting relies on a combination of indirect targeting methods and microelectrode recordings (MER).^9^, ^10^ The indirect targeting methods do not take into account the variability in the location of VIM.^11^, ^12^ This may result in heterogeneous and suboptimal outcomes with tremor surgery.^13^, ^14^, ^15^ Post hoc analysis of active DBS electrode or thermal lesion location with probabilistic^12^, ^16^, ^17^ and deterministic^18^, ^19^ tractography links successful surgical outcomes with the modulation of the cerebellar input to the VIM nucleus (or the dentate‐rubro‐thalamic tract [DRT]). Currently there are no methods for targeting DRT, and individualized tractography‐based VIM targeting (T‐VIM) remains desirable in functional neurosurgery.

Probabilistic tractography provides objective structural connectivity measures and better resolution for crossing fibers.^20^ This approach is computationally intensive and ideally suited for research investigations. Notably, the commonly used probabilistic (eg, FMRIB software library (FSL), statistical parametric mapping (SPM), MRtrix) and the advanced deterministic tractography software (with a better resolution for crossing fibers, eg, 3D‐Slicer) are not approved for clinical applications. The intention of this study was to develop a method for T‐VIM that targets for tremor surgery that can be used clinically and is easy to execute and integrate with stereotactic targeting software. This method allows the VIM localization in relation to the pyramidal tract (PT) and the medial lemniscus (ML). These tracts define the lateral and posterior boundaries of the VIM nucleus. We compared the T‐VIM coordinates with the conventional targeting methods in ET patients and a cohort of healthy controls. In a separate cohort of surgical tremor patients, we compared the coordinates of T‐VIM with intraoperative coordinates of surgical VIM target (S‐VIM). To study the robustness of the T‐VIM localization in the surgical cohort, we further investigated its structural connectivity with a commonly used probabilistic tractography software (FSL, FMRIB v. 5.0.1 software library, www.fmrib.ox.ac.uk/fsl).

## Methods

The study procedures were approved by the institutional research ethics board.

### Imaging Cohort

This study included 18 patients with ET. Two independent movement disorder neurologists confirmed the diagnosis of medically refractory ET (failure of at least 2 medications). From an imaging database of 60 healthy controls, we included controls in their 5th and 6th decades of life (n = 22).

### Operative Cohort

From November 2014 to January 2015, 6 patients underwent microelectrode‐guided VIM thalamotomy or DBS implantation at our center. The patients were deemed good surgical candidates by a multidisciplinary team. In this cohort, there was an equal number of ET patients (n = 3) and tremor‐dominant Parkinson's disease patients (n = 3). The targeting for the surgical cohort was done using conventional methods based on indirect targeting. T‐VIM tractography, target identification and comparison to S‐VIM and MER results was performed separately, in a post hoc manner.

#### Imaging Protocol and Preprocessing

Structural and diffusion weighted (60 directions of diffusion gradient) images were acquired on 3T MRI scanners. Eddy current and movement artifact corrections were applied. StealthViz software (v1, Medtronic Inc., Minneapolis, Minnesota) was used for tensor calculation. This software uses a deterministic tractography and is integrated with the stereotactic targeting software used for tremor surgery. The diffusion weighted and structural MRI images were coregistered, and the accuracy of coregistration was verified. Detailed methodology of the preprocessing pipeline is provided in the supplementary section.

### T‐VIM Identification

#### Region of Interest (ROI) and Tractography for PT and ML

**Figure 1:**
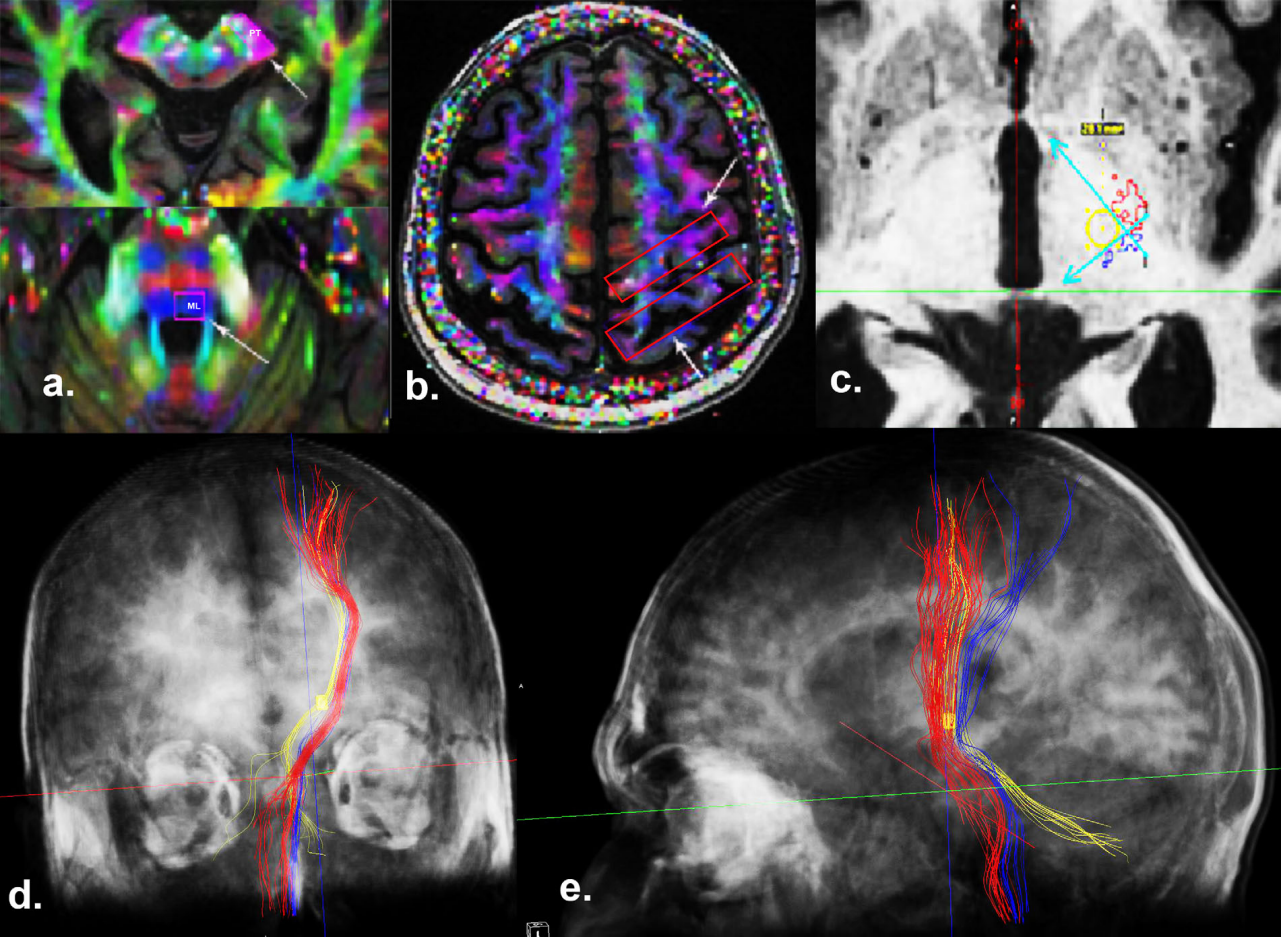
The methodology of tracking the pyramidal tract (PT), the medial lemniscus (ML), and the dentate‐rubro‐thalamic tract (DRT). **a and b**: The regions of interest (ROIs) were placed at the cerebral peduncle and primary motor cortex for tracking the PT. Similarly, ROIs were placed at the dorsal brain stem and sensory cortex for visualizing ML. **c**: To define the lateral and posterior borders of tractography‐based–ventral intermediate nucleus (VIM), we created 2 lines at the medial border of the PT (red) and the anterior border of the ML (blue) at the anterior commissural‐posterior commissural level. We then generated a tractography‐based‐VIM ROI (a 3‐mm circle was first drawn at the angle of the PT and ML, and the thalamic ROI was then placed with its center coinciding with the center of the circle). The projections of this ROI were visualized, keeping the tracking angle constant at 60°. **d**: Coronal view of the 3‐dimensional reconstruction (semitransparent scalp and skull), demonstrating the relationship between the PT (red), ML (blue), and DRT and thalamo‐cortical projections from the VIM (yellow). The bilateral cerebellar contribution to the DRT can also be visualized. **e**: Sagittal view of the 3‐dimensional reconstruction (semitransparent scalp and skull), demonstrating the motor cortical projections from the VIM ROI (yellow) anterior to the ML (blue).

The ROI location of the PT and ML tracking was chosen using the fractional anisotropy (FA) color map (Fig. 1).^21^ For PT, ROIs were placed over the cerebral peduncle and the ipsilateral primary motor cortex. Similarly for ML, the ROIs were placed over the dorsal column at the levels of the brain stem and the primary sensory cortex. The tracking angles were set at 45° and 60° for PT and ML, respectively. The other tracking parameters were a FA stop value of 0.2 and a seed density of 1. Typically PT and ML are considered easy to track. Therefore in cases where these major tracks were not trackable we chose not to proceed with the imaging analysis.

#### Determination of the T‐VIM and Its Structural Connections

Anatomically PT and ML, respectively, represent the lateral and the posterior ventral boundaries of the VIM thalamus. To delineate the T‐VIM, the images were aligned in the anterior commissural (AC)‐posterior commissural (PC) plane and an axial slice at the AC‐PC level was selected. We placed a cubic thalamic ROI (length 4.6 [0.4] mm, width 3.8 [0.5] mm, height 5.4 [0.6] mm, volume 93.5 [15] mm^3^) with its center equidistant (3 mm) from the borders of the PT and ML (Fig. 1). The distance of 3 mm was deemed a clinically safe margin to avoid motor and sensory side effects associated with either lesioning or DBS. The tracking parameters were the same for the PT and ML, with a tracking angle of 60^0^. Finally, tractography was performed to explore the structural connections of T‐VIM.

### Outcome Measures

For the imaging cohort, we calculated the location of the center of the VIM ROI relative to the midline, the lateral wall of the third ventricle, and the posterior commissure. These measurements were compared with the most commonly used Guiot's method for indirect targeting.^22^, ^23^ According to this method, the VIM is located between the second and third 12th of the AC‐PC length in front of the PC and 11.5 mm from the lateral wall of the third ventricle (or 15 mm from the midline) at the AC‐PC level. Using this method, Benabid et al. reported the mean DBS electrode tip location at 21.6% (SD 0.7) of the AC‐PC length in front of the PC.^22^

For the surgical cohort, we calculated the Euclidian distance between the S‐VIM and the T‐VIM. We also assessed the accuracy of diffusion tensor imaging (DTI)‐based targeting by comparing the location of the sensory thalamus (VC), ML, PT, and VIM efficacious zone with the MER findings. The location of the VC was inferred on the MER by the presence of tactile (deep or superficial) response and stimulation‐induced (low threshold current = 5‐10 μA; frequency = 200 Hz, duration = 1 second, pulsewidth = 0.3 seconds) localized paresthesias. Similarly, ML was inferred by the stimulation‐induced (high threshold current = 50‐100 μA, frequency = 200 Hz, duration = 1 second, pulsewidth = 0.3 seconds) hemibody paresthesias. With these stimulation parameters, the current spread is limited to within 1 mm of the microelectrode tip.^24^ The presence of muscle contraction or dysarthria upon stimulation was ascribed to the stimulation of the PT. The optimal target zone within VIM was defined by the presence of kinesthetic cells, tremor cells, and most importantly tremor reduction (either tremor arrest or visible tremor reduction) on intraoperative stimulation. For correlation to the physiological data, the three tracts (PT, ML, and DRT) were visualized on the structural T1 (with a multiplanar reconstruction length of 1 mm, similar to the 1‐mm isovoxel of the 3T structural T1 scan) and exported to the stereotactic planning software (Framelink, Medtronic Inc.). For the purposes of tractography, we defined VC as the thalamic area with sensory projections at or above the level of the AC‐PC plane. Similarly, the sensory projections below the AC‐PC plane were interpreted as ML. Finally, the primary motor cortical projections traversing the lateral boundary of the thalamus were defined as PT.

### Statistical Analysis

Statistical analysis was performed with SPSS (v.22, IBM Corp., Armonk, NY). We compared continuous variables with analysis of variance and categorical variables with a chi‐squared test with *P* < .05 defined as statistically significant.

## Results

### Imaging Cohort

#### Comparison of T‐VIM Coordinates With Conventional Targeting Method

**Lateral Coordinate. T**he distance of the T‐VIM from the lateral wall of the 3rd ventricle was 12.5 [1.2] mm and 12.4 [1.1] mm for cases and controls, respectively (Table 1). This coordinate was more lateral than the commonly used targeting coordinates (11.5 mm from the ventricle wall) both in ET patients (*P* = .0004) and controls (*P* = .0002). However, the distance from the midline was similar for each method for the ET patients (15‐mm conventional coordinate vs 15.03 [1.3] mm, *P* = .99), but significantly different for controls (15‐mm conventional coordinate vs 13.5 [1.4] mm for T‐VIM, *P* = .00005). This difference could be accounted for by the wider 3rd ventricle diameter in ET patients (5.7 [1.94] mm vs 3.1 [1.86] mm, *P* = .001).

**Anterior Coordinate. T**he T‐VIM derived coordinate was more anterior (relative to PC) in both the ET patients and healthy controls when compared with the conventional targeting coordinates. The distance of the T‐VIM from PC was 8.5 [1.1] mm in the ET patients and 7.9 [1.1] mm in the controls (*P* = .15). When expressed in terms of percentage of intercommissural length, the anterior coordinate was approximately 32% of the intercommissural distance anterior to the PC.

**Table 1:** Comparison of diffusion tensor imaging (DTI)–based ventral intermediate nucleus targeting (T‐VIM) and conventional targeting method (S‐VIM)

	Conventional indirect targeting	DTI based targeting in ET patients, n = 14; M (SD)	DTI based targeting in controls, n = 15; M (SD)	Comparison of conventional targeting with DTI‐based targeting in ET patients^a^	Comparison of DTI‐based targeting in ET patients and controls^a^
Anterior coordinate					
Proportion of AC‐PC length	21.6%	32% (4%)	32% (3.6%)	.00005	.99
Distance from PC (mm)	6.7 (0.3)	8.5 (1.1)	7.9 (1.1)	.00005	.15
Lateral coordinate					
Distance from midline (mm)	15	15 (1.3)	13.5 (1.4)	.99	.006
Distance from third ventricle wall (mm)	11.5	12.5 (1.2)	12.4 (1.1)	.0004	.82

AC, anterior commissure; PC, posterior commissure; ET, essential tremor.

a *P* values are reported for comparison.

#### T‐VIM and Its Projections

The projections from the T‐VIM were trackable in only 14 ET patients (26 hemispheres) and 15 healthy controls (29 hemispheres). The components of these projections are discussed below.

**DRT.** The DRT projections from the contralateral cerebellar hemisphere were visualized in 16 of the 26 hemispheres in ET patients, and in 3 additional hemispheres the DRT projections reached up to the decussation of the superior cerebellar peduncles. In all of the hemispheres, apparent ipsilateral fibers to the DRT were observed, which is likely an artifact arising because of the inability of single‐tensor tractography^27^ and tractography in general^28^ to properly visualize crossing fibers (see the supplementary material for a description of the tractography algorithm). Similar results were obtained for the control group (contralateral contribution in 16 hemispheres and 3 additional hemispheres with projections to the decussation of superior cerebellar peduncles).

**Cortical Projections. C**onsistent projections to the primary motor cortex were visualized both in the ET patients and controls (25 of 26 hemispheres in ET patients and all 29 hemispheres in controls). Projections to the premotor cortex were also visualized in a few instances (2 of 26 hemispheres in ET patients and 4 of 29 hemispheres in controls).

### Confirmation of T‐VIM Projections With Probabilistic Algorithm

For each of the 6 patients, we were able to identify connectivity between the T‐VIM and the motor/premotor area as well as the ipsilateral and contralateral dentate nucleus, thus confirming the previous findings from a deterministic algorithm (Supplementary Fig. 2). The probabilistic algorithm was effective in identifying crossing fibers with a robust contralateral cerebellar contribution to DRT in all patients.

**Figure 2:**
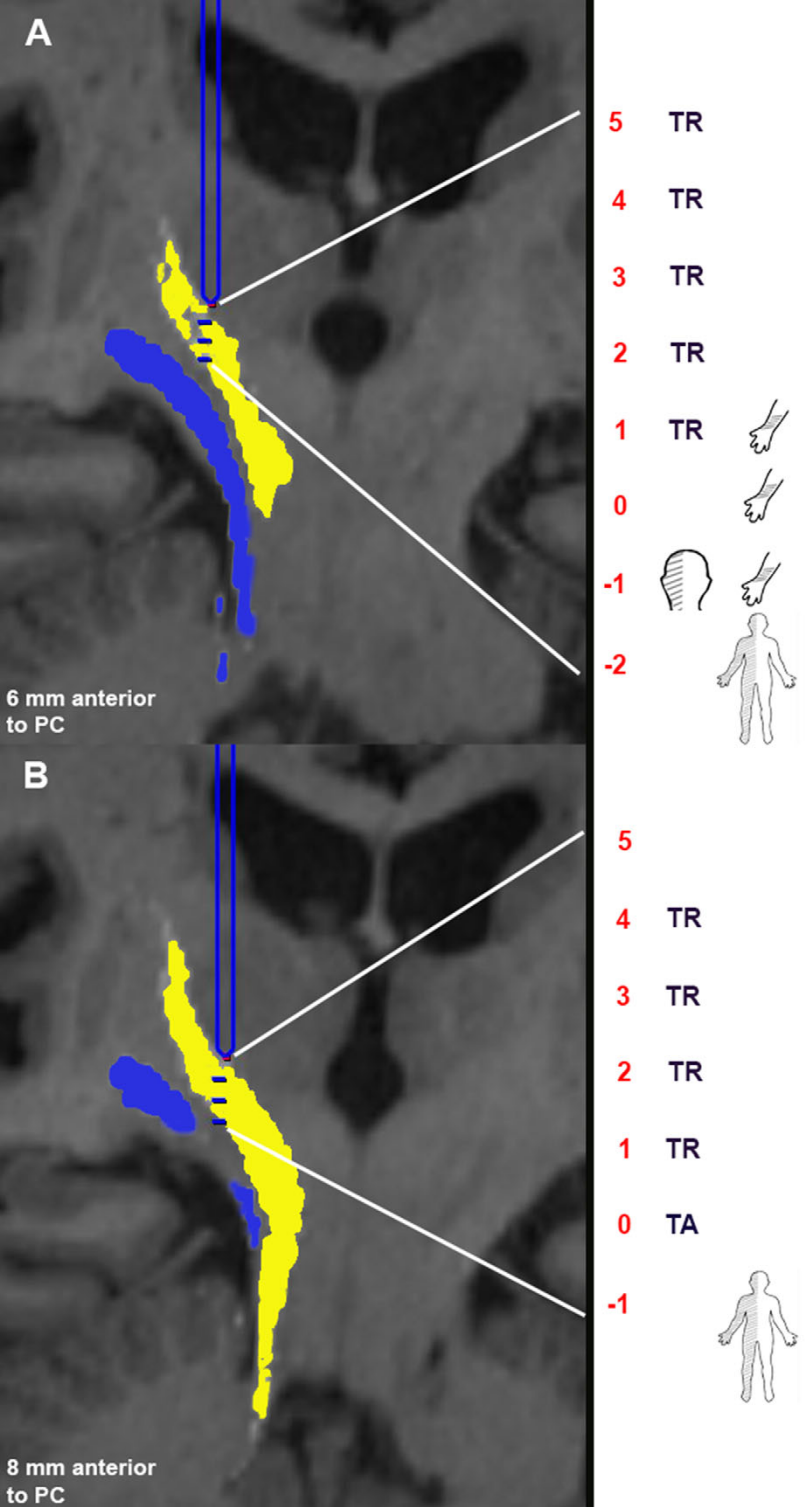
Projection views from a single patient (P1) in the operative cohort with tractography images overlaid in the stereotactic planning software. The dentate‐rubro‐thalamic tract (DRT) is color coded in yellow and ML in blue. The findings from the intraoperative stimulation and testing are summarized in the anatomical sketches on the right side. The level of posterior commissural (PC) was selected as Z = 0, and the recordings proximal to it are depicted as positive, and those below the PC level were depicted as negative. **A**: The first trajectory (S1) is 6 mm anterior to the PC (2 mm behind the tractography‐based target). The tip of the electrode (blue) is localized at the proximal level of tremor reduction (5 mm above the target). The distal end (interrupted blue line) projects to the total recorded length (2 mm beyond the target). We observed tremor reduction (TR) for a 4‐mm distance starting at the superior edge of the DRT. The sensory thalamus was identified 1 mm above target (the border between the DRT and the medial lemniscus [ML]). The ML was identified 1 mm beyond the target corresponding to the DRT ML border below the anterior commissural‐posterior commissural plane. **B**: Second trajectory (S2) at the tractography‐based target (8 mm anterior to the PC). The tip of the microelectrode recordings electrode (blue) is again localized at the proximal level of the tremor reduction (5 mm above the target). The distal end (interrupted blue line) projects to the total recorded length (1 mm beyond target). We observed tremor reduction (TR) for a 5‐mm distance starting at the superior edge of the DRT. The ML was identified 1 mm beyond the target corresponding to the electrode entry into the ML. The DBS electrode was implanted in the second trajectory S2.

### Operative Cohort

We performed 9 microelectrode trajectories in 6 patients (mean age 71.7 [5.9] years, Clinical Rating Scale for Tremor (CRST) subscale B score 17.8 [5.4]) undergoing either DBS (n = 2) or thalamotomy procedures (n = 4).The mean T‐VIM coordinates were *X* = 14.3, *Y* = 7.2, and *Z* = 0.3. The Euclidian distance between the S‐VIM and the T‐VIM was 1.6 (1.1) mm. The final chosen target was modified in 5 of 6 patients based on MER. In all of these cases, T‐VIM would have accurately predicted the direction of intraoperative adjustment. Both the T‐VIM and the S‐VIM were located anterior to the first trajectory in 4 patients and posterior in 1 patient. In 1 procedure, the S‐VIM coincided with the T‐VIM in the first trajectory itself. At 1 year, there was a 58.9% reduction in tremor scores on the side treated with thalamotomy (mean score preop 19.2 [5.2] vs post op score 7.8 [2.3], *P* = .002) (Supplementary Table 1).

#### Correlation With MER—Sensory and Motor Findings

VC was identified with MER in 5 patients, and its location correlated with the border of the DRT and the ML at or above the AC‐PC plane (at the border in 2 trajectories and slightly anterior to it in 3 trajectories, mean 0.5 [0.7] mm anterior to the border). Electrophysiologically, ML was identified in 6 patients below the AC‐PC plane (mean 2 [1.2] mm below PC) at DRT and ML border (mean 0.03 [1.3] mm anterior to the border). None of the patients reported motor side effects upon microelectrode stimulation. The MER tip was >3mm from the PT border in all MER trajectories.

The trajectory view from a single patient (P1T1) is shown in Figure 2, with the tip of the probe indicating the proximal site of tremor efficacy and the dashed line corresponding to the total length of the trajectory (7 mm, starting 5 mm above and 2 mm below the target). The tractography projections in the stereotactic planning software (Framelink, Medtronic Inc.) are color coded (ML blue and DRT yellow) for ease of interpretation. The putative MER trajectory corresponding to the T‐VIM (P1T2, 8 mm anterior to posterior commissure) had a longer zone of tremor efficacy (5 mm total with tremor arrest at the target). ML was identified at the bottom of this trajectory.

#### Correlation With MER—Tremor Efficacy Findings

**Figure 3:**
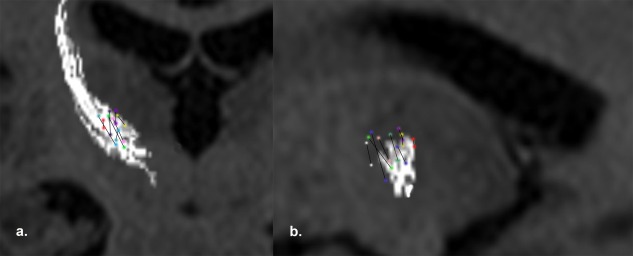
The normalized trajectory angles (2 colored circles joined by a line) with tremor reduction are plotted in relation to the dentate‐rubro‐thalamic tract (DRT) and the thalamocortical projections of the ventral intermediate nucleus on reconstructed images from T1 axials, with a) coronal (7mm anterior to the posterior commissure) and b) sagittal (12 mm from the ventricular wall). The reduction in tremor was observed for variable lengths (3‐5 mm). The trajectories are dispersed within DRT. (P1T1: pink, P1T2: green, P2T1: red, P2T2: blue, P3T1: purple, P4T1: yellow, P5T2: bottle green, P6T1: navy blue). One additional track (P5T1: white) with no tremor efficacy was anterior to the DRT.

The mean length of the thalamus with stimulation‐induced tremor was 3.8 [1.2] mm. The proximal and distal ends of the region of tremor suppression were within the DRT margins (proximal end 0.4 [1.2] mm below the superior margin, 1.4 [1.5] mm medial to the medial margin, and 2.8 [1.7] mm anterior to the posterior margin; distal end 2.5 [1.7] mm superior to the inferior margin, 3.5 [1.4] mm medial to the medial margin, and 1.8 [1.2] mm anterior to the posterior margin). The normalized trajectory angles from all of the surgical patients are shown in coronal (7 mm in front of PC) and sagittal (12 mm from ventricle wall) projections in Figure 3. The efficacious zone within the DRT was distributed across the entire course of DRT. We failed to observe tremor efficacy in 1 MER trajectory (P5T1, 6mm anterior to PC). A subsequent MER trajectory (P5T2, 4mm anterior to PC) demonstrated significant efficacy. Assessment of the location of these MER tracts with respect to the DRT demonstrated accurate localization of P5T2 within DRT (green trajectory in Fig. 3).

## Discussion

We report a tractography‐based targeting method for the VIM nucleus for tremor surgery. The coordinates of the T‐VIM were more anterior and lateral to the conventional indirect targeting method. In this validation study, T‐VIM imaging accurately correlated with electrophysiology and clinical testing. Based on these results, T‐VIM targeting appears to be a reliable and accurate method for VIM targeting.

The VIM nucleus is approximately 4 × 4 × 6 mm in size, bordering laterally with the PT and posteriorly with the VC.^9^, ^29^, ^30^, ^31^ It receives fibers from the dentate nucleus through the contralateral superior cerebellar peduncle.^32^, ^33^ The VIM nucleus is a relay nucleus and projects to M1, although a minority of fibers also terminate in the premotor cortex.^23^, ^29^, ^34^, ^35^, ^36^ We used this anatomical knowledge to develop a methodology of tractography‐based targeting of the VIM nucleus. Using the PT and the ML as internal landmarks, an ROI for the T‐VIM was created similar to the anatomical size of this thalamic subnucleus and identified its structural connectivity with the cerebellum and the motor cortex. This methodology yields similar results, both location and connectivity of T‐VIM, in ET patient and controls. When the imaging results were compared with intraoperative MER, we observed good accuracy in predicting the location of the S‐VIM, the VC, the ML, and the PT. Overall, the T‐VIM target is more lateral and anterior than the conventional targeting method. Anthofer and colleagues recently compared the atlas‐based VIM target with the location of the DRT (as determined by deterministic tractography) in a cohort of tremor patients.^18^ They concluded that the optimal site for DBS implantation was more lateral than the planned atlas‐based trajectory.

Several groups have reported loss of efficacy in the long term after thalamic DBS or thalamotomy.^13^, ^14^, ^15^ Disease progression and stimulation tolerance are the 2 potential explanations.^14^, ^15^ However, targeting error may also be responsible for poor efficacy.^8^ Dysfunction of the cerebello‐thalamo‐cortical network (or tremor network) is implicated in tremor pathogenesis.^37^, ^38^, ^39^, ^40^, ^41^, ^42^, ^43^ More important, targeting the tremor network is considered crucial for successful outcomes after VIM DBS or thalamotomy.^16^, ^17^ Klein and colleagues compared the connectivity of the efficacious DBS contacts with the adjacent nonefficacious contacts in patients undergoing VIM DBS.^16^ They reported that efficacious DBS contacts have significantly higher anatomical connectivity with the contralateral cerebellum and ipsilateral motor cortex, implying a successful modulation of the entire tremor network. The authors logically envisioned a tractography‐based stereotactic targeting for effectively targeting this network in the future.

**Figure 4:**
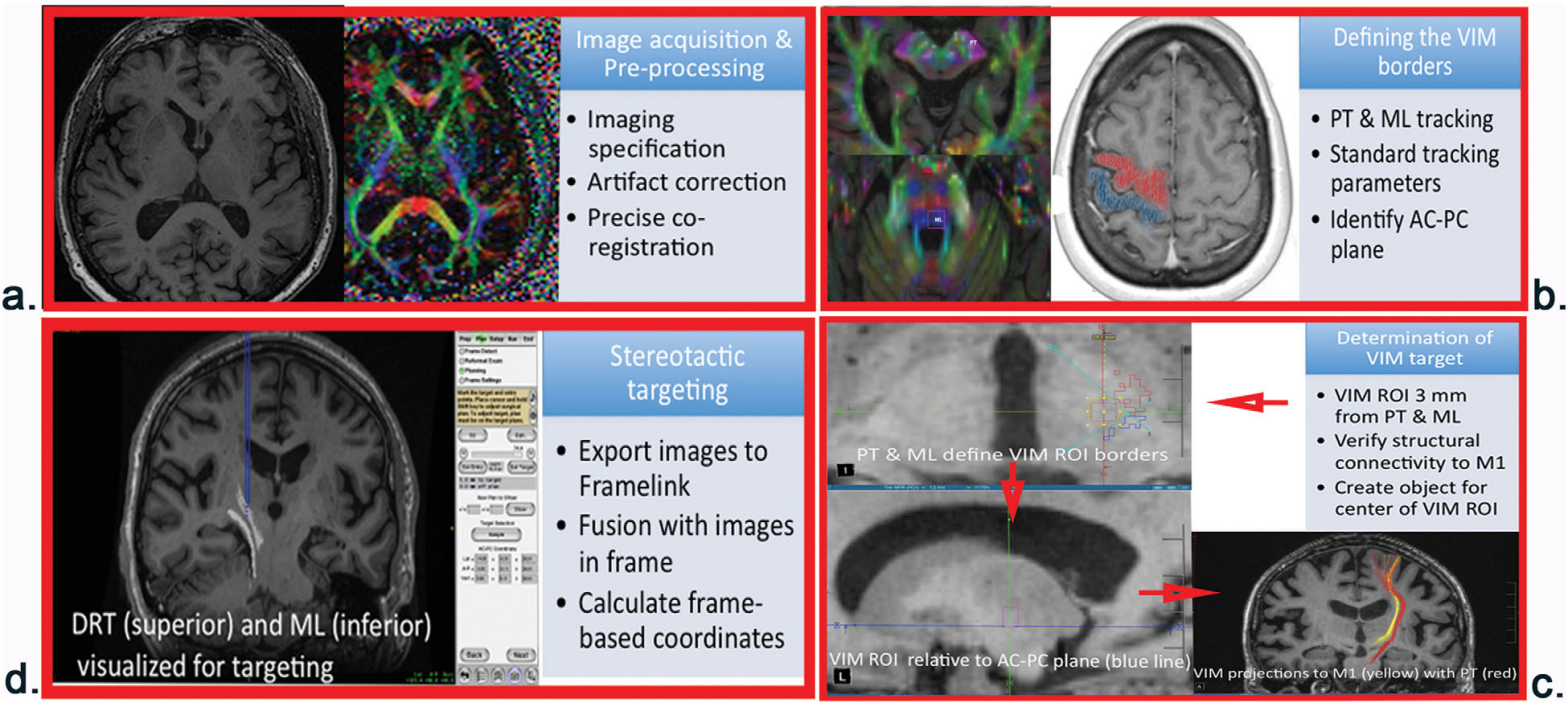
Summarized description of tractography‐based ventral intermediate nucleus methodology, listing the necessary steps for tract delineation and target calculation. The specific components of each step are specified within each box. PT, Pyramidal tract; ML, medial lemniscus; AC, anterior commissure; PC, posterior commissure; VIM, ventral intermediate nucleus; ROI, region of interest; DTI, diffusion tensor imaging.

Previous studies have used post hoc DTI analysis to delineate structural connections of the efficacious DBS contacts or lesions in order to define the imaging predictors of outcome.^19^, ^44^ When considering a T‐VIM as a targeting approach, 2 important issues need to be addressed: whether the methodology is anatomically valid and reproducible and whether it improves the anatomical accuracy over current methods of VIM targeting. We performed a stepwise quality‐control process to avoid errors from inadequate imaging, motion artifact, and inaccurate coregistration between structural imaging and DTI (Fig. 4). The method of deriving the VIM location relative to the PT and ML differs from previous approaches, such as those identifying VIM based on the connectivity to premotor and motor areas.^12^, ^25^, ^26^ The precise identification of the PT and the ML with this approach allowed us to localize an efficacious target while avoiding major side effects, especially motor side effects resulting from the transgression of PT. The structural connections of T‐VIM were also screened to ensure accurate localization. Using this methodology, the Euclidian distance between the T‐VIM and the S‐VIM was 1.6 (1.1) mm. We also found a <1 mm concordance between imaging findings and the intraoperative localization of the VC, the ML, the PT.

Although a detailed discussion about the merits and limitations of deterministic and probabilistic tractography is beyond the scope of this study, we gained important insights by comparing these 2 approaches in the surgical cohort. We chose a streamlined algorithm‐based deterministic tractography because of its ease of analysis and seamless integration with the stereotactic targeting software. This method has intrinsic limitations for visualizing crossing fibers.^27^ For example, we noticed the presence of an ipsilateral cerebellar contribution to DRT in all of the patients and controls. In contrast, the decussating cerebellar fibers were better visualized with the probabilistic tractography (with a ball‐and‐stick algorithm). Probabilistic tracking builds a connectivity distribution between a predefined seed structure and every other voxel in the brain based on the most probable diffusion direction. As an example, we analyzed 5000 samples from each voxel in the seed region to build a quantitative measure of the probability of connectivity with other voxels in the brain. This methodology generated more connected regions than the streamline approach, similar to the observations of others.^45^ However, for the purposes of stereotactic targeting, the higher structural connectivity obtained by the probabilistic methods does not provide a more accurate target definition. Furthermore, probabilistic algorithms may not be as efficient as streamline tractography in reconstructing long pathways.^46^ This limitation arises because of a decrease in connectivity from a higher uncertainty in orientation during fiber propagation.^47^ Finally, probabilistic algorithms are computationally intensive, require considerable processing time, and are not approved for clinical applications. Overall, this comparison demonstrated that the methodology and principles of deterministic tractography for tract and target definitions are more relevant for clinical applications. In addition, the fact that deterministic tractography limits the number of connected regions and provides greater visualization of distal fibers provides an important measure of assurance to clinicians, who must evaluate the DTI images to ensure they are suitable for targeting. Therefore, at the present time we recommend the use of deterministic algorithms for this purpose. However, clinically approved deterministic tractography platforms with advanced algorithms for tensor calculation and fiber propagation are desirable in the future.

### Study Limitations

The resolution of the DTI sequences available for clinical application can certainly be improved for stereotactic neurosurgery.^48^ For example, the DTI voxel size was not isotropic (0.94 × 0.94 × 3 mm), which could potentially decrease precision in the *Z*‐axis. However, the increase in resolution with novel imaging protocols has to be balanced with an increase in scan time, especially for patients with movement disorders (ET and PD), limiting their capability to stay still.^49^ Therefore, we chose an imaging protocol with a short scanning time (25 minutes combined time for both structural and diffusion‐weighted scans) without creating undue patient discomfort. Similar to previous work by Klein and colleagues,^16^, ^50^ we used 60 directions of diffusion gradients to optimize the consistent and reproducible tracking of the DRT.^51^ The choice of placing T‐VIM ROI 3 mm medial and anterior to PT and ML is not purely arbitrary. Most thalamotomy lesions have a diameter of 4 to 6 mm. Therefore, a distance of 3 mm from the PT and the ML would allow the lesion to be safely placed within the T‐VIM without producing deficits from the lesioning of these tracts. Similarly for DBS, a 3‐mm distance from the electrode tip would safely allow for sufficient titration of stimulation amplitude (up to 3‐5 volts) for a clinically efficacious tremor control without side effects from PT and ML stimulation. With this targeting methodology, the *Z*‐coordinate of the T‐VIM was fixed at the AC‐PC plane because the ventral border of thalamus coincides with the AC‐PC plane.^52^ The superior border of the T‐VIM ROI was 5 mm proximal from the AC‐PC plane corresponding with the supero‐inferior dimensions of human VIM.

The ET patients and controls in our study were not age‐matched (mean age of ET patients 69.6 [5.9] years vs 58.6 [6.2] years for controls). Previous studies have shown that the FA value for PT reaches a plateau around 40 years of age and declines slowly afterward without a clear cut‐off.^53^ Although we chose an older group of healthy controls, the FA differences related to different age profiles of the two groups needs to be taken into account. The surgical cohort included patients with ET and PD. Although the quantitative diffusion tensor parameters may differ between these two diseases, it does not reach the threshold to alter the tract appearance. Overall since tract depiction is not significantly different this methodology can be used to image T‐VIM regardless of pathophysiology.

## Conclusion

We propose a novel method for T‐VIM targeting. The methodology is accurate, and useful. The total time for generating the data is less than 1 hour per patient allowing for greater applicability. Future studies are required to optimize T‐VIM targeting and assess the long‐term clinical efficacy. With increasing use of non‐invasive approaches for tremor surgery such as MRgFUS and gamma knife, we foresee this methodology become increasingly important to facilitate and optimize targeting and compensate for the lack of direct physiological validation associated with these procedures.

## Author Roles

1. Research Project: A. Conception, B. Organization, C. Execution; 2. Statistical Analysis: A. Design, B. Execution, C. Review and Critique; 3. Manuscript Preparation: A. Writing the First Draft, B. Review and Critique.

F.S.: 1A, 2A, 2B, 2C, 3B

V.K.: 1A, 2A, 2B, 2C, 3B

N.K.K.K.: 1A, 2B, 2C, 3B

A.L.: 1A, 3B

M.H.: 1A, 3B

## Full financial disclosures of all authors for the past 12 months

Francesco Sammartino MD = no financial disclosures

## Supporting information

Additional Supporting Information may be found in the online version of this article at the publisher's web‐site.

Supplementary Information

Supplementary Information
