# Supplementary figures and images for: Tractography‐Based Ventral Intermediate Nucleus Targeting: Novel Methodology and Intraoperative Validation
Source: Mov Disord. 2016 May 23;31(8):1217–25. doi: 10.1002/mds.26633 (PMC5089633; doi:10.1002/mds.26633)

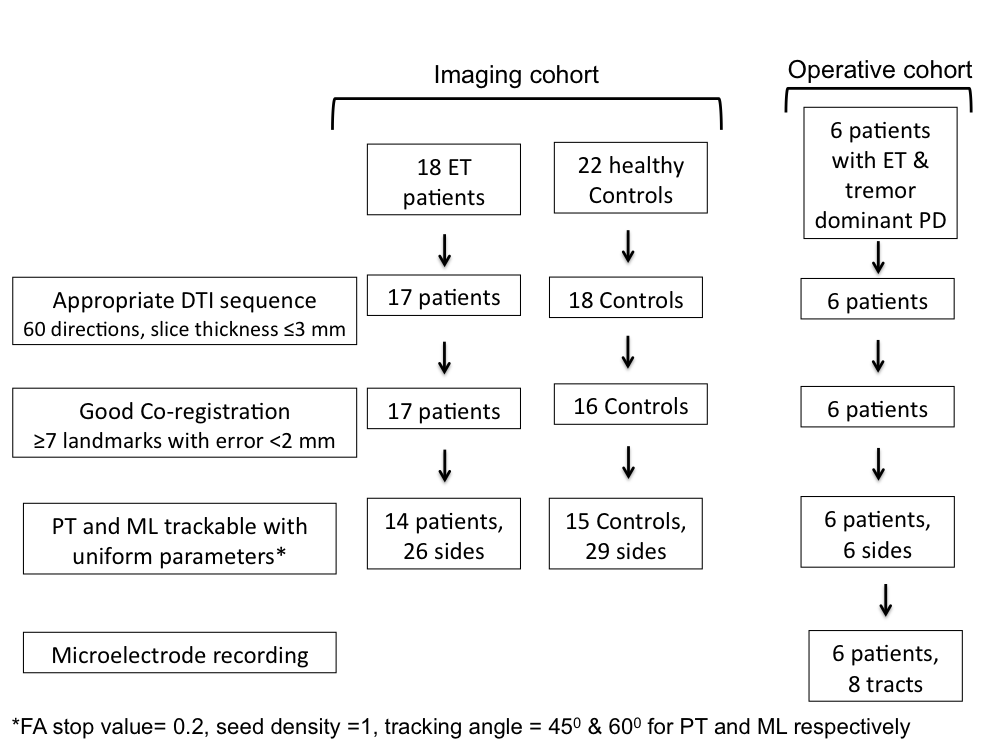

Supplement: Supplementary file 1 — Supplementary Information [file MDS-31-1217-s001.tif]
