# Supplementary material for: Tractography‐Based Ventral Intermediate Nucleus Targeting: Novel Methodology and Intraoperative Validation
Source: Mov Disord. 2016 May 23;31(8):1217–25. doi: 10.1002/mds.26633 (PMC5089633; doi:10.1002/mds.26633)
Supplement: Supplementary file 2 — Supplementary Information [file MDS-31-1217-s002.doc]

Methods –

DTI Imaging protocol

MRI scans were acquired on a 3T GE MRI in both patients and controls. The DTI (Echo Planar/Spin Echo sequence) for the imaging cohort controls and operative patients (both ET and tremor dominant PD patients) was acquired using an 8-channel phased-array head coil (GE Signa, WI, USA), with 60 directions of diffusion gradients (b = 1000 s/mm^2^; 0.94 x 0.94 x 3 mm voxel size; T-Echo = 86.6 ms; T-Repetition = 12000 ms; matrix = 128 x 128, FOV= 23.0, 64 slices). As part of the imaging protocol T1-weighted 3D FSPGR axial images were also acquired (0.85 x 0.85 x 1mm voxel size, 256 x 256 matrix, FOV = 220 mm, T-Echo = 5 ms, T-Repetition = 12 ms, TI = 300 ms).

Imaging for the ET patients in imaging cohort was similarly acquired on a 3T GE MRI (GE Discovery, WI, USA). The DTI parameters included a 60 gradients (1.8 x1.8 x 2 mm voxel size; T-Repetition = 8000 ms; b=1000s/mm2; matrix = 128x128) and a T1-weighted 3D FSPGR (voxel size 1.2x1.2x0.93 mm, 256x256 matrix, FOV = 240mm, T-Echo = minimum, TI = 450 ms).

Preprocessing pipeline

Prior to the analysis, all MRI images were converted from DICOM to Nifti format using the dcm2nii software (<http://www.mccauslandcenter.sc.edu/mricro/mricron/dcm2nii.html>). Correction for eddy-current and movement artifacts was first applied using the FMRIB toolbox, a part of the FSL software (v5.0.7, <http://fsl.fmrib.ox.ac.uk/fsl/>). The T1 and DTI images were then processed using the Brainsuite software platform (<http://brainsuite.org/processing/>). The software includes an all-in-one solution for skull stripping of the T1-weighted image, non-uniformity compensation, and creation of a mask to be used for correction of B0 field inhomogeneity artifacts. The diffusion images were co-registered to the anatomical T1-weighted image using the INVERSION method and were corrected for susceptibility-induced distortions using a modified implementation of the non-rigid registration method.

Images were then converted back to the DICOM format using in-house software and imported into the Stealth Planning Station (Medtronic Inc., MN, USA). The tensors calculations were performed using the StealthViz software (v1, Medtronic Inc., Minneapolis, MN, USA) using the tractography algorithm based on the deterministic single tensor tractography. This tractography platform is integrated with the stereotactic planning software (Framelink v 5, Medtronic Inc., MN, USA).

Coregistration of tractography and structural MR images

For each subject, the DTI images were registered to the T1-weighted structural scan using StealthViz software (v1, Medtronic Inc., Minneapolis, MN, USA). We verified the accuracy of the coregistration by comparing the location of 10 different anatomical landmarks in the proximity to the thalamus (anterior commissure, posterior commissure, bilateral superior colliculi, bilateral body of fornix, bilateral superior cerebellar peduncle, and the posterior and inferior borders of the splenium of the corpus callosum in the midsagittal plane). The registration was considered adequate if the maximum distance in the location of 7 out of 10 landmarks was less than 2mm between the T1 and diffusion-weighted images.

*Delineation of T-VIM structural connections with probabilistic tractography*

We performed probabilistic tractography for patients in the ‘surgical cohort’ group since this group was a part of the operative validation study. The diffusion images were preprocessed using FMRIB v.5.0.1 software library. Briefly, we applied eddy current correction and aligned all diffusion directions to a reference b0 image. The b0 image was then linearly registered to the structural T1 after brain extraction[^3^](#_ENREF_3), and finally the diffusion tensors were fitted. Following this, the diffusion parameters were sampled for each voxel using FSL ‘bedpostX’.[^4^](#_ENREF_4) We then performed probabilistic tractography (FSL ‘probtrackX’) from within each patient’s diffusion space. Seed masks, corresponding the size of the original T-VIM ROI DICOM overlay were created in the T1 space and we obtained transformation matrices between the two coordinate spaces. In order to have an adequate comparison of tractography parameters we chose the following parameters - 5000 samples; curvature threshold, 0.2; no waypoint, exclusion, or termination masks and no advanced options. The total processing time for one subject was 24 hours on a Macbook Air laptop (Core i5, 4GB RAM). We visually inspected the output from ‘probtrackX’ as overlay in T1 space.

Operative details

The technical details of the surgical procedure for thalamotomy and thalamic DBS have already been described elsewhere.[^5^](#_ENREF_5) The VIM was targeted based on the methodology described previously and the individual tracts (PT, ML and DRT) were then exported to the stereotactic planning software (Framelink v.5, Medtronic Inc., Minneapolis, MN). For the surgical cohort, we also created a cubic object (from the Segmentation window of the StealthViz software) corresponding to the voxel at the center of the VIM ROI and generated a DICOM overlay on the anatomical T1. This DICOM dataset was then rigidly aligned to the preoperative frame-based T1 used for targeting using the Framelink software (Medtronic Inc., Minneapolis, MN).

On the day of surgery each patient underwent positioning of the Leksell frame under local anesthesia followed by a 1.5T (GE Signa Excite) MRI scan (T1 3D FSPGR, voxel size = 1.01x1.01x1.4 mm, matrix = 256x256, TE= minimum). The operating surgeon authors (AL or MH) chose the frame-based coordinates for the Vim target on the day of surgery using the conventional formulaic method. We also calculated the frame coordinates of the tractography-based target. Our technique for microelectrode recording has been described elsewhere.[^6^](#_ENREF_6) Briefly we started MER 10 mm above the target, in order to map the VIM, VIM/VC border, and medial lemniscus. We performed microelectrode stimulations at 1 mm increments to assess tremor efficacy. The first ‘run’ of microelectrode recording was undertaken at the target chosen by conventional method. Intraoperative adjustments were then made based on the findings of MER recordings and side effect profile on microelectrode stimulation (current – 5-100 μA, frequency - 200 Hz, duration - 1 s, pulsewidth - 0.3 s). These findings were incorporated in planning the final trajectory for DBS lead implantation or creation of a thermal lesion.

Results –

*Accuracy of imaging registration and tractography*

We were able to successfully perform VIM tractography in 14 out of the 18 (77.8%) ET patients. One patient was excluded due to missing images from the imaging dataset. With the tracking parameters described above, ML could not be tracked in 3 other patients (mean age 74.3±9.8 years, mean disease duration 36.3±24.1 years and CRST- subscale B score 27.7±3.1). Of the 14 ET patients, PT and ML could only be tracked in one hemisphere in 2 additional patients. The mean age of the patients included in the imaging cohort was 69.6±5.9 years with 3 females, 11 males (mean disease duration 35.8±14.7 years, CRST subscale B score 25.2±5.7). There are no significant differences between patients included in the imaging cohort and those that were excluded (p values for comparison between age, disease duration and CRST-B are 0.5, 0.98 and 0.48 respectively).

15 healthy controls (29 sides, mean age - 58.6±6.2; 11 females, 4 males) were included in the final imaging analysis. Two patients in the control group were excluded due to poor accuracy of coregistration between the structural and DTI images (less than 7 anatomical landmarks with coregistration error <2 mm). The mean error in alignment was 1.5±0.9 mm for ET patients and 1.6±1.2 mm for the control group (p=0.8). We observed consistent discrepancy in the alignment of the anterior commissure, likely originating from the susceptibility effect from air-filled sphenoid sinuses despite being minimized by our pre-processing.[^7^](#_ENREF_7)

Figure legends –

Supplementary Figure.1 The flow diagram of study patients. In the imaging cohort, 77.8% of ET patients and 68.2% of controls initially enrolled were utilized after the quality control steps with respect to adequacy of the imaging studies, ability to track PT & ML and good coregistration between tractography and structural imaging. The MER tractography findings were correlated in 6 additional patients undergoing microelectrode guided thalamic procedures (DBS or thalamotomy) for their refractory tremor (3 with ET and 3 with tremor-dominant PD).

Supplementary Figure. 2 Comparison of deterministic (streamline) and probabilistic tractography for the surgical cohort patients.

Top panel (A-E) – Represents the 3D projections of PT (red), ML (blue) and DRT (green) obtained using deterministic tractography. The tracts are overlaid on the preoperative axial T1 MR images. The T-VIM ROI is also shown (pink square), in all cases encompassing the DRT fibers.

Panels 2-4: G-L (axial), M-R (coronal) and S-X (sagittal) views of T-VIM projections obtained by probabilistic tractography methods and overlaid on structural T1 MR images. It can be visualized (G-L) that the structural connectivity of T-VIM is wider, and more irregularly shaped compared with deterministic methods (A-F). Similarly coronal (M-R) and sagittal (S-X) images demonstrate a number of fibers that may or may not directly correspond to DRT, but have been delineated with the probabilistic method. This affects the visualization of tracts, since the anatomical pathway of DRT is not immediately discernible (see green projections, panels M, N, U, V, X)

On the coronal projections (M-R) the red arrow indicates the presumed contralateral component of the cerebral projections to T-VIM (DRT). On the sagittal projections (S-X) the red line indicates the position of the central sulcus. The T-VIM to ipsilateral M1 was obtained in all 6 patients.

Supplementary Table 1. Tremor scores (scale from 0-4) on the treated side in the surgical cohort at baseline and 1 year after surgery. Individual sub-scores have been added to reflect the total score in the specific body region

| Tremor score | Patient 1 | | Patient 2 | | Patient 3 | | Patient 4 | | Patient 5 | | Patient 6 | |
| --- | --- | --- | --- | --- | --- | --- | --- | --- | --- | --- | --- | --- |
|  | Pre | 1 yr | Pre | 1 yr | Pre | 1 yr | Pre | 1 yr | Pre | 1 yr | Pre | 1 yr |
| Face | 0 | 0 | 0 | 0 | 0 | 0 | 0 | 0 | 0 | 0 | 0 | 0 |
| Tongue | 0 | 0 | 0 | 0 | 0 | 0 | 0 | 0 | 0 | 0 | 1 | 1 |
| Voice | 0 | 0 | 0 | 0 | 0 | 0 | 0 | 1 | 2 | 1 | 1 | 1 |
| Head | 1 | 1 | 1 | 0 | 0 | 0 | 0 | 0 | 1 | 0 | 2 | 2 |
| RUE | 5 | 1 | 5 | 0 | 6 | 0 | 4 | 0 | 5 | 0 | 2 | 3 |
| LUE | 3 | 5 | 3 | 3 | 4 | 4 | 3 | 4 | 4 | 4 | 4 | 1 |
| Trunk | 0 | 0 | 1 | 0 | 0 | 0 | 0 | 0 | 0 | 0 | 0 | 0 |
| RLE | 0 | 0 | 1 | 0 | 0 | 0 | 1 | 0 | 2 | 0 | 0 | 0 |
| LLE | 0 | 0 | 1 | 0 | 0 | 0 | 0 | 0 | 3 | 3 | 0 | 0 |
| Orthostatic | 0 | 0 | 0 | 0 | 0 | 0 | 1 | 0 | 0 | 0 | 0 | 0 |
| Handwriting | 2 | 0 | 3 | 1 | 3 | 1 | 1 | 1 | 3 | 1 | 3 | 1 |
| Drawing | 2 | 0 | 3 | 1 | 3 | 1 | 1 | 1 | 4 | 1 | 3 | 1 |
| Pouring | 3 | 0 | 4 | 0 | 3 | 0 | 0 | 0 | 4 | 1 | 3 | 1 |
| Total score | 16 | 7 | 22 | 5 | 19 | 6 | 11 | 7 | 28 | 11 | 19 | 11 |

References –

1. Bhushan C, Haldar JP, Joshi AA, Shattuck DW, Leahy RM. INVERSION: A robust method for co-registration of MPRAGE and Diffusion MRI images. Joint Annual Meeting ISMRM-ESMRMB; 2014; Milan, Italy.

2. Bhushan C, P. HJ, A. JA, M. LR. Correcting Susceptibility-Induced Distortion in Diffusion-Weighted MRI using Constrained Nonrigid Registration. Asia-Pacific Signal & Information Processing Association Annual Summit and Conference

; 2012; Hollywood, CA, USA.

3. Behrens TE, Johansen-Berg H, Woolrich MW, et al. Non-invasive mapping of connections between human thalamus and cortex using diffusion imaging. Nat Neurosci 2003;6(7):750-757.

4. Behrens TE, Berg HJ, Jbabdi S, Rushworth MF, Woolrich MW. Probabilistic diffusion tractography with multiple fibre orientations: What can we gain? Neuroimage 2007;34(1):144-155.

5. Kumar R, Lozano AM, Sime E, Lang AE. Long-term follow-up of thalamic deep brain stimulation for essential and parkinsonian tremor. Neurology 2003;61(11):1601-1604.

6. Levy R, Lozano AM, Hutchison WD, Dostrovsky JO. Dual microelectrode technique for deep brain stereotactic surgery in humans. Neurosurgery 2007;60(4 Suppl 2):277-283; discussion 283-274.

7. Mukherjee P, Chung SW, Berman JI, Hess CP, Henry RG. Diffusion tensor MR imaging and fiber tractography: technical considerations. AJNR American journal of neuroradiology 2008;29(5):843-852.
